# Delays in the Stroke Care Pathway in a Low-Income Setting: An Audit Study from Mozambique

**DOI:** 10.3390/ijerph22071008

**Published:** 2025-06-26

**Authors:** Helena Buque, Lee Smith, Dino Lopes, Damiano Pizzol, Elder Lorenzo, Nachan Arroz, Lazara Bacallau, Mohsin Sidat, Evangelina Namburete Bauaze, Hipólito Nzwalo

**Affiliations:** 1 Neurology Department, Maputo Central Hospital, Maputo 1100, Mozambique; elderlorenzo31@gmail.com (E.L.); nachancadmiela@yahoo.com.br (N.A.); bacallaolazara33@gmail.com (L.B.); 2 Community Health Department, Faculty of Medicine, Eduardo Mondlane University, Maputo 1100, Mozambique; mmsidat@gmail.com; 3 Ageing and Cerebrovascular Research Group, ABC Research Institute, Faculty of Medicine and Biomedical Sciences, University of Algarve, 8005-139 Faro, Portugal; 4 Centre for Health, Performance and Wellbeing, Anglia Ruskin University, Cambridge CB1 1PT, UK; lee.smith@aru.ac.uk; 5 Emergency Department, Maputo Central Hospital, Maputo 1100, Mozambique; dilopes938@gmail.com; 6 Health Unit, Eni, Maputo 1100, Mozambique; damianopizzol8@gmail.com; 7 Faculty of Health Sciences, Catholic University, Beira 821, Mozambique; evanamburete@gmail.com

**Keywords:** stroke care, treatment delays, low income, Mozambique

## Abstract

**Background:** The burden of stroke is on the rise in low-income countries (LICs). Organized stroke care (OSC) is crucial for improving outcomes in LICs and is the very first step to reducing delays in diagnosis and treatment. We aim to evaluate delay times (DT) in accessing OSC at the national reference hospital of Mozambique, a LIC from southern Africa. **Methods**: An observational study based on consecutive case series of 59 stroke patients confirmed by computed tomography (CT) scans over a period of 3 months (May–July 2023). The total DT (from stroke onset to inward hospitalization) was the main outcome. Other specific DTs were analyzed including initial symptoms to arrival and admission (DT0), arrival to CT scans (DT1), arrival of laboratory results (DT2), and arrival to inward hospitalization (DT3). **Results:** The mean age was 61.9 (min 30–max 90) and 45.8% were female. The median total DT was 20 h. The median time DT0 was 10.6 h (interquartile range (IQR): 16.48). The median DT1 and DT2 were 4 h (IQR: 3.5) and 5 h (IQR: 2.6), respectively. The median DT3 was 10 h (IQR: 4). None of the patients were treated under a stroke code. **Conclusions:** This study reveals an unacceptable prehospital and in-hospital DT. Waiting for the CT scan contributed to a large proportion of the total DT, which among other factors can be explained by the absence of a stroke code and limited imaging capacity. These findings mirror disparities in stroke care seen in other LICs, where late presentation, scarce imaging, and limited specialized protocols are common. The urgent implementation of organized prehospital and in-hospital stroke pathways is needed in Maputo to improve outcomes.

## 1. Introduction

Stroke is a leading cause of death worldwide and the third leading cause of premature death and disability, representing the leading neurological cause of disability-adjusted life years (DALYs), accounting for 4.1% of the global total [1,2].

The phrase “Time is Brain” emphasizes that every minute without treatment leads to a loss of neurons at a rate of approximately 120 million per hour—equivalent to 3.6 years of brain aging [3].

The pathway from symptom onset to definitive care significantly affects stroke survival and outcomes [4,5]. Organized stroke services—such as dedicated stroke units and protocols for rapid assessment—consistently reduce mortality, complications, and disability, and improve functional recovery [6]. However, these services are found in 91% of high-income countries and in only 18% of low-income countries, demonstrating a marked global inequity [7]. International guidelines stress the importance of public education on stroke risk factors, the establishment of specialized care units, and coordinated emergency response [7,8,9].

In Sub-Saharan Africa, where stroke fatality rates can reach approximately 40%, studies have demonstrated that establishing organized stroke care decreases mortality from 27.7% to 7.2% and significantly lowers the rate of complications [10,11,12,13,14,15]. Mozambique is one of the Sub-Saharan countries with a high burden of stroke. For instance, in the national referral hospital, the “Maputo Central Hospital” (MCH), at least three patients with acute stroke are hospitalized per day, with an estimated in-hospital fatality of 22,7% and with a 28-day case fatality of 33.3% to 49.6% [16,17]. Despite these alarming figures, the country lacks formal pathways and specialized stroke care teams.

This study aims to evaluate delay times (DTs) in accessing organized stroke care services among patients at MCH. By analyzing specific segments of the care pathway, we seek to highlight system gaps, inform strategic interventions, and align with best practices from comparable LIC settings.

## 2. Materials and Methods

A retrospective case series study was conducted over a three-month period (May to July 2023) involving patients admitted with stroke to the adult emergency department of MCH in Mozambique. The clinical records of all stroke patients who presented to the emergency department during this period were reviewed. Demographic data, including age, sex, and place of origin were collected. Additional information was extracted regarding the time of arrival at the emergency department, the triage priority assigned, and the time intervals between arrival and the delivery of key medical services. This included the initial assessment by the triage physician, the emergency or observation physician, as well as the timing of laboratory tests, imaging procedures, and admission to inpatient wards. Arrival time was primarily determined using the registration timestamp recorded by the administrative staff. In cases where this time was missing, the time documented by the first nursing assessment was used as a proxy.

### Statistical Analysis

Statistical analysis was conducted using R Commander software (version 4.3.2). A univariate descriptive analysis was performed for all variables, with the results presented in the form of graphs, maps, and tables. For numerical variables, descriptive statistics included the mean, median, standard deviation, coefficient of variation, range, minimum, and maximum. For categorical variables, absolute and relative frequencies were calculated. Associations between variables were tested using Pearson’s Chi-square test or Fisher’s exact test, as appropriate. A *p*-value of less than 0.05 was considered statistically significant.

## 3. Results

### 3.1. Socio-Demographic Profile

Of the 130 patients hospitalized with a diagnosis of stroke during the study period, 38 (29%) were excluded due to unavailable clinical records, and 33 (35%) were excluded for not meeting the inclusion criteria of having undergone diagnostic imaging prior to discharge. A total of 59 patients met the eligibility criteria and were included in the analysis. The patient’s age ranged from 30 to 90 years, with a mean age of 62 years. Of these, 32 (54%) were male and 27 (46%) were female. Regarding the source of referral, 51 (86%) arrived from home, 6 (11%) were transferred from general hospitals, and 2 (3%) were referred from health centers. Among those who came from home, the majority, 32 (54%), lived between 10 and 25 km from the emergency department, while 14 (24%) resided between 5 and 10 km away.

### 3.2. Time from Symptom Onset to Arrival at the Emergency Department

Of the 59 patients evaluated, 12 (20%) had no documented time of symptom onset upon arrival at the emergency department. Among the remaining patients, 17 (29%) sought emergency care between 12 and 24 h after symptom onset, and 12 (20%) presented between 10 and 12 h.

**Figure 1:**
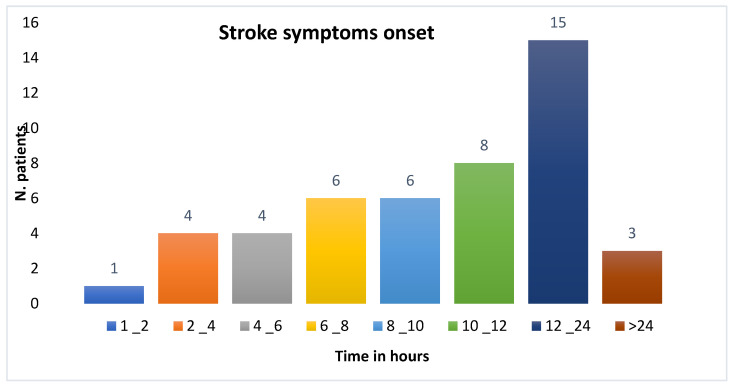
The distribution of patients according to the time of the evolution of stroke symptoms.

Only, five patients (8%) arrived within 4 h of symptom onset and three (5%) patients presented more than 24 h after symptom onset. The median time from symptom onset to hospital arrival was 10 h (interquartile range [IQR]: 16.48), as shown in Figure 1.

The lowest number of stroke admissions occurred on Mondays and Fridays, with three patients (5%) each. The highest number of admissions was recorded on Tuesdays and Wednesdays, with 12 patients (20%) each, followed by Thursdays and Saturdays, which each accounted for 10 patients (17%).

### 3.3. Waiting Time and Priority Assessment in the Emergency Department

Of the 59 patients, 34 (57%) were assigned the highest priority level (red), 14 (24%) received an orange or yellow priority, and 11 (19%) had no priority color recorded upon arrival at the emergency department (ED). Triage was performed using the Manchester Triage System, in which colored labels (red, orange, yellow, green, and blue) indicate the severity and urgency of the patient’s condition. The time from ED arrival to the hospitalization ward ranged from 5 to 24 h, with variation observed across different days of the week. The longest average waiting times were recorded on Mondays (15 h), Thursdays (13 h), and Saturdays (11 h). The overall mean time spent in the ED before hospitalization was 9 h.

**Figure 2:**
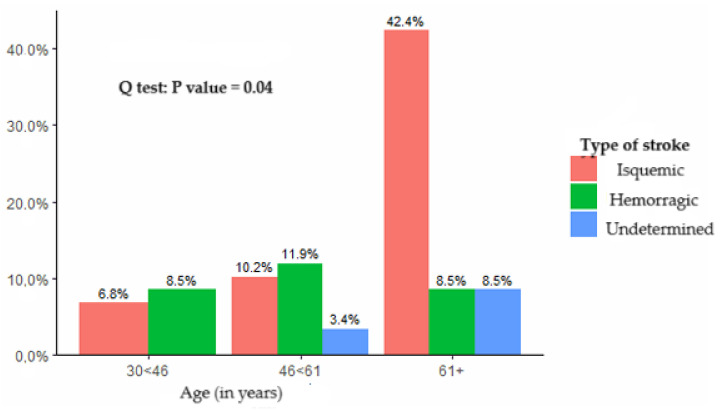
The type of stroke experienced by the patients under study.

Regarding stroke classification, 35 patients (59%) had ischemic strokes, 17 (29%) hemorrhagic strokes, and in 7 patients (12%), the stroke type was not determined at the time of admission. Ischemic strokes were more frequently observed in patients over 60 years of age, while hemorrhagic strokes were more common among younger patients, as shown in Figure 2.

Twenty-one patients (36%) received laboratory tests within 2 to 3 h of arrival, while twelve patients (20%) received them between 3 and 5 h. For 14 patients, the time of laboratory test initiation was not recorded.

**Figure 3:**
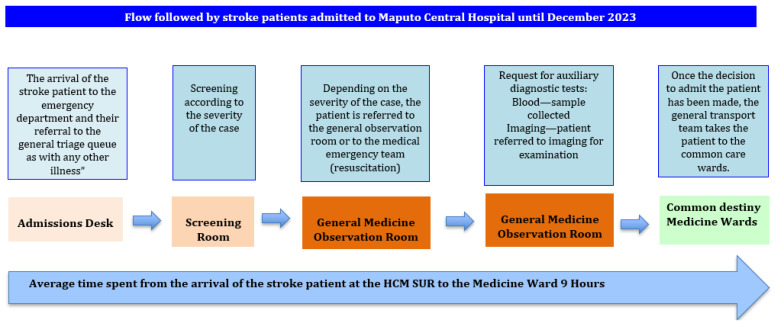
The flowchart followed by stroke patients at the emergency department.

CT scans were performed more than 300 min (5 h) after arrival in 16 patients. Additionally, 11 patients underwent CT scans at between 180 and 300 min (3 to 5 h), 7 patients at between 120 and 180 min (2 to 3 h), and 6 patients at between 30 and 120 min. In 19 cases the CT scan time was not documented. The median time from arrival at the emergency department (ED) to CT scan completion was 4 h (IQR: 3.5). Regarding admission destinations, 52 patients (88%) were admitted to the internal medicine wards, 5 (8%) to the neurology department, and 1 (2%) to the ICU and intermediate care, as shown in Figure 3.

## 4. Discussion

Our audit reveals critical delays in both prehospital and in-hospital phases of stroke care at MCH. The median total DT reached 20 h, with significant delays in prehospital (median DT0 = 10.6 h) and in-hospital CT access times (DT1 = 4 h). Moreover, one-third of patients lacked CT imaging, and a similar proportion had incomplete clinical records, illustrating major resource and documentation gaps. These delays strongly undermine timely stroke diagnosis and treatment.

A small proportion of patients presented within the critical time window for acute reperfusion therapies, typically within 4.5 h. Access to timely stroke care is shaped by a combination of local and regional organizational, educational, cultural, socioeconomic, geographic, and healthcare system factors [18,19,20]. Poor public knowledge of stroke symptoms, and the urgency of seeking care often lead to late hospital arrivals. Transport challenges, including limited ambulance availability and poor road infrastructure, further increase prehospital time. Within the hospital, the limited availability of CT scanners and trained stroke personnel delay diagnosis and treatment initiation.

This delay is also found in other Sub-Saharan African nations where there is limited recognition of stroke symptoms and transport barriers are a reality [5,7,10,15,18,19,20]. Similar patterns have been reported in Nigeria, Ethiopia, and Uganda, where median times from symptom onset to hospital presentation range between 12 and 48 h [21,22,23,24,25]. When patients arrive, in-hospital delays are exacerbated by limited CT availability. Mozambique has fewer than one CT scanner per million inhabitants. Although the MCH is equipped with two CT scanners, it does not have stroke-specific protocols—such as a stroke code—to expedite brain imaging in suspected stroke cases [4,5,6,10,26]. Even in high-resource countries, fewer than 5% of acute stroke patients arrive within the window necessary to receive reperfusion therapy, such as intravenous thrombolysis [27]. Supportive interventions such as blood pressure control, anticoagulation reversal, and the early management of complications are essential to improving patients’ outcomes [28,29]. With a mean total delay of 20 h, the timely delivery of these interventions to our stroke patients was not achieved.

Similar patterns are seen in Ethiopia where only around 14% of patients reached the hospital within 4.5 h and half received a CT scan [30] and in Ghana, where in-hospital delays frequently exceed recommended targets, with the median time to CT interpretation being almost 17 h [31,32]. In Burkina Faso, the median onset-to-door time was about 7 h, with only 19% arriving within the thrombolysis window [26]. Other studies in LICs show that timely and appropriate acute stroke treatment is feasible and reduces the complications and risk of death [20,33]. Although reperfusion therapies are not currently available at our hospital, the implementation of other timely supportive measures remains feasible and necessary to improve prognosis.

Regarding the reasons for the delay in arriving at the emergency service, they were not studied in this study. However, in several studies carried out in countries in Sub-Saharan Africa, multiple factors contribute to these delays—a lack of awareness of stroke symptoms, living far from the hospital, poor access roads, limited public transportation, and a lack of ambulance services are the main reasons for the delay in arriving at the hospital [21,22,23,24]. While our study did not include stakeholder interviews, previous research in Ghana, Nigeria, and Zambia suggests that patients often first consult traditional healers or delay care due to fatalistic beliefs and social stigma [34,35,36].

At the hospital level, we observed that although most patients were triaged with high urgency (red or orange) at admission, the in-hospital pathway did not match the recognized emergency of stroke; for example, with an unacceptable median time to CT scan (4 h) and an average time to laboratory results (3 h). These times greatly exceed international recommendations, which suggest CT imaging should occur within 25 min and lab results within 45 min of arrival [37]. Furthermore, over 85% of patients were admitted to the internal medicine wards rather than neurology units. Stroke is a neurological emergency that requires coordinated care from a specialized multidisciplinary team including neurologists and stroke-trained staff, to limit neurological injury, prevent clinical deterioration, and optimize recovery outcomes [38,39,40,41,42]. The implementation of organized stroke care is not only clinically beneficial but also cost-effective and essential in resource-limited settings [20].

This study has several limitations. The retrospective nature of the study, the elevated number of patients excluded because of missing or incomplete information, and the absence of patient and provider perspectives all limit the completeness of the analysis and the interpretation of hospital delay times. Because the study was conducted at the national referral hospital located in the country’s capital, selection bias must be considered, particularly the potential underrepresentation of patients from peripheral or rural areas or those with less severe clinical presentations. Indeed, in a different clinical context, we found that access to diagnostic brain imaging at MCH was limited to only a small fraction of patients presenting severe symptoms [43].

Multivariate analysis to identify the predictors of delay times was not carried out due to the small sample size. In addition, data on key potentially explanatory factors—such as the mode of hospital arrival and the specific causes of delays, including cognitive and cultural perceptions of stroke—were not collected. These gaps highlight important directions for future research. Community education campaigns about stroke symptoms, the development of prehospital stroke response systems, and the training of healthcare staff are cost-effective strategies successfully implemented in LICs. Hospital-based improvements such as establishing stroke units, implementing CT and laboratory workflow protocols, and forming multidisciplinary stroke teams also yield better patient outcomes [4,5,6,10,26].

This study was not designed to test a novel hypothesis, but rather to document and bring visibility to the current state of stroke care in a low-income setting where systematic data are rarely collected or published. The findings underscore critical gaps in acute stroke management and highlight the urgent need for structural improvements. Based on our observations, we believe that the most pressing actions to improve stroke outcomes in this context include the establishment of dedicated stroke units, the training of frontline healthcare workers in early stroke recognition and emergency response, the implementation of public awareness campaigns to promote timely care-seeking behavior, and the development of referral systems that enable patients to reach appropriate care facilities within the therapeutic window. Addressing these issues is essential to reduce stroke-related mortality and disability in Mozambique and in similar low-resource settings.

## 5. Conclusions

This audit revealed an unacceptable pre- and intrahospital delay time for stroke care in Mozambique’s major referral hospital, an essential first step in addressing system inefficiencies. However, that our study had several limitations means we cannot fully capture patient, provider, or policy perspectives. Nevertheless, the education of patients, the training of professionals, and the establishment of a stroke chain of care should be priorities.

The establishment of a dedicated stroke unit will enable a multidisciplinary and integrated approach, with a specialized team capable of providing personalized and high-quality care.

## Data Availability

The original contributions presented in this study are included in the article. Further inquiries can be directed to the corresponding author.
